# The neural processing of foreign-accented speech and its relationship to listener bias

**DOI:** 10.3389/fnhum.2014.00768

**Published:** 2014-10-08

**Authors:** Han-Gyol Yi, Rajka Smiljanic, Bharath Chandrasekaran

**Affiliations:** ^1^SoundBrain Lab, Department of Communication Sciences and Disorders, Moody College of Communication, The University of Texas at AustinAustin, TX, USA; ^2^UT Sound Lab, Department of Linguistics, College of Liberal Arts, The University of Texas at AustinAustin, TX, USA; ^3^Institute for Neuroscience, The University of Texas at AustinAustin, TX, USA

**Keywords:** foreign-accented speech, speech perception, fMRI, implicit association test, neural efficiency, primary auditory cortex, inferior frontal gyrus, inferior supramarginal gyrus

## Abstract

Foreign-accented speech often presents a challenging listening condition. In addition to deviations from the target speech norms related to the inexperience of the nonnative speaker, listener characteristics may play a role in determining intelligibility levels. We have previously shown that an implicit visual bias for associating East Asian faces and foreignness predicts the listeners' perceptual ability to process Korean-accented English audiovisual speech (Yi et al., 2013). Here, we examine the neural mechanism underlying the influence of listener bias to foreign faces on speech perception. In a functional magnetic resonance imaging (fMRI) study, native English speakers listened to native- and Korean-accented English sentences, with or without faces. The participants' Asian-foreign association was measured using an implicit association test (IAT), conducted outside the scanner. We found that foreign-accented speech evoked greater activity in the bilateral primary auditory cortices and the inferior frontal gyri, potentially reflecting greater computational demand. Higher IAT scores, indicating greater bias, were associated with increased BOLD response to foreign-accented speech with faces in the primary auditory cortex, the early node for spectrotemporal analysis. We conclude the following: (1) foreign-accented speech perception places greater demand on the neural systems underlying speech perception; (2) face of the talker can exaggerate the perceived foreignness of foreign-accented speech; (3) implicit Asian-foreign association is associated with decreased neural efficiency in early spectrotemporal processing.

## Introduction

Foreign-accented speech (FAS) can constitute an adverse listening condition (Mattys et al., 2012). Perception of FAS is often less accurate and more effortful compared to native-accented speech (NAS; Munro and Derwing, 1995b; Schmid and Yeni-Komshian, 1999; Van Wijngaarden, 2001). The reduced FAS intelligibility has been attributed to deviations from native speech in terms of segmental (Anderson-Hsieh et al., 1992; Van Wijngaarden, 2001) and suprasegmental (Anderson-Hsieh and Koehler, 1988; Anderson-Hsieh et al., 1992; Tajima et al., 1997; Munro and Derwing, 2001; Bradlow and Bent, 2008) cues. Nevertheless, listeners can adapt to FAS following exposure or training (Clarke and Garrett, 2004; Bradlow and Bent, 2008; Sidaras et al., 2009; Baese-Berk et al., 2013). Thus, listener's perception of FAS perception can improve over time (Bradlow and Pisoni, 1999; Bent and Bradlow, 2003). The neuroimaging literature on FAS perception is scant. However, perception of foreign phonemes has been shown to engage multiple neural regions. These include the superior temporal cortex, which matches the auditory input to the preexisting phonological representations (“signal-to-phonology mapping”) in the articulatory network, encompassing the motor cortex, inferior frontal gyrus, and the insula (Golestani and Zatorre, 2004; Wilson and Iacoboni, 2006; Hickok and Poeppel, 2007; Rauschecker and Scott, 2009). In particular, the inferior frontal gyrus exhibits phonetic category invariance, in which the response patterns differ according to between-category phonological variances but not to within-category acoustical variances (Myers et al., 2009; Rauschecker and Scott, 2009; Lee et al., 2012). Accordingly, processing of artificially distorted speech which, reduces speech intelligibility but does not necessarily introduce novel phonological representations, has been shown to involve additional recruitment of the superior temporal areas, the motor areas, and the insula, but not the inferior frontal gyrus (for review, see Adank, 2012). These findings lead to two predictions regarding neural activity during FAS processing. First, lack of adaptation to FAS would manifest in increased activity in the superior temporal auditory areas, due to the increased demand on auditory input processing. The primary auditory cortex is sensitive not only to rudimentary acoustic information such as frequency, intensity, and complexity of the auditory stimuli (Strainer et al., 1997), but also to the stochastic regularity in the input (Javit et al., 1994; Winkler et al., 2009) and attention (Jäncke et al., 1999; Fritz et al., 2003). The response patterns of the primary auditory cortex is modulated by task demands (attentional focus: Fritz et al., 2003; target properties: Fritz et al., 2005), attention, training effects (frequency discrimination: Recanzone et al., 1993), and predictive regularity in the auditory input (Winkler et al., 2009). Furthermore, early acoustic signal processing time for speech stimuli has been shown to be reduced with accompanying visual information, indicating that the primary auditory cortex activity is modulated by crossmodal input (Van Wassenhove et al., 2005). Thus, the primary auditory cortex is attuned to analyzing the details of the incoming acoustic signals, but is also influenced by contextual information and modulated by experience. Second, difficulty in resolving phonological categories would manifest in increased activity in the articulatory network. In contrast to the early spectrotemporal analyses of speech, later stages of phonological processing are largely insensitive to within-category acoustic differences and exhibit enhanced sensitivity to across-category differences. Such phonological categorization is achieved via a complex network involving the inferior frontal cortex, insula, and the motor cortex (Myers et al., 2009; Lee et al., 2012; Chevillet et al., 2013).

**Figure 1:**
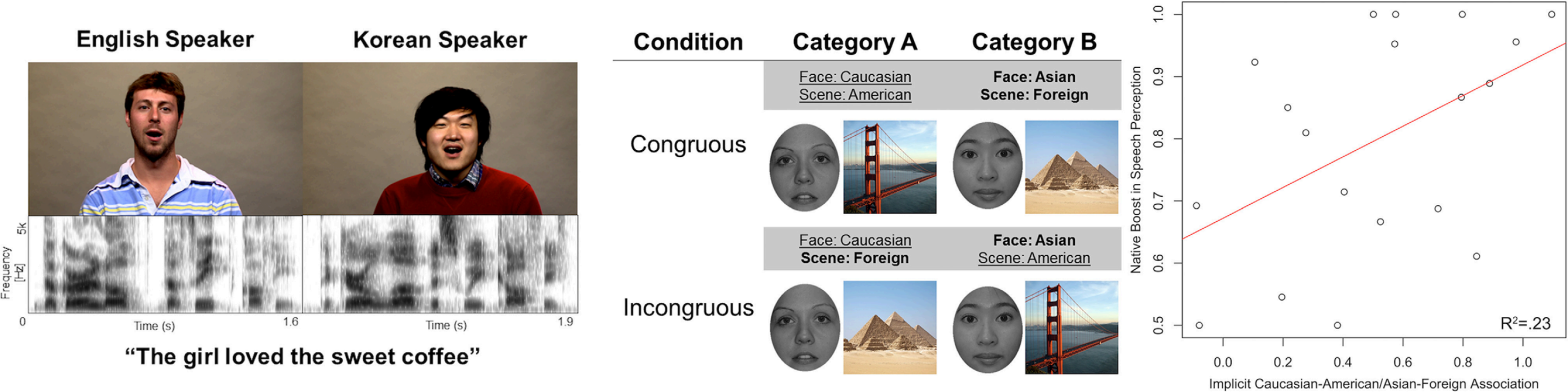
**Left:** Example stimuli for native-accented and foreign-accented speech. **Center**: Example stimuli and the design schematic for the implicit association test. **Right**: Implicit Asian-foreign association was associated with relative foreign-accented speech perception difficulties when faces were accessible to the listeners (figures adapted from Yi et al., 2013).

Signal-to-phonology mapping, however, is not the only factor that modulates FAS perception. Listener beliefs regarding talker characteristics have been shown to modify the perceptual experience of speech (Campbell-Kibler, 2010; Drager, 2010). Specifically, different assumptions held about speaker properties by the individual listeners can potentially alter perception of the otherwise identical speech sounds. For instance, explicit talker labels (e.g., Canadian vs. Michigan) and indexical properties (e.g., gender, age, socioeconomic status) implied in visual representation of the talkers can change phonemic perception for otherwise identical speech sounds, even when the listeners are aware that this information is not accurate (Niedzielski, 1999; Strand, 1999; Hay et al., 2006a,b; Drager, 2011). The impact of perceived talker characteristics on FAS perception can be complex. Explicit labels have been linked to increased response times in lexical tasks for FAS, thus indicating increased processing load (Floccia et al., 2009), while visual presentation of race-matched faces have been shown to increase intelligibility for Chinese-accented speech (McGowan, 2011). These findings suggest that listener variability in FAS intelligibility may be partly accounted for using measures of listeners' susceptibility to these indexical cues (Hay et al., 2006b). In social psychology, the implicit association test (IAT) has been used extensively to quantify the degree of implicit bias which may not be measured using explicit self-reported questionnaire entries (Greenwald et al., 1998, 2009; Mcconnell and Leibold, 2001; Bertrand et al., 2005; Devos and Banaji, 2005; Kinoshita and Peek-O'leary, 2005). During an IAT, the participants are instructed to make associations between two sets of stimuli (e.g., American vs. Foreign scenes; Caucasian vs. Asian faces). The response times between two conditions (e.g., Caucasian-American and Asian-Foreign vs. Caucasian-Foreign and Asian-American) are compared, and the magnitude of the difference between the mean RTs are considered to reflect the degree of implicit bias toward the corresponding association. In non-speech research domains, the IAT measures have been shown to be positively correlated with neural responses to dispreferred stimuli in various networks, including the amygdala, prefrontal cortex, thalamus, striatum, and the anterior cingulate cortex (Richeson et al., 2003; Krendl et al., 2006; Luo et al., 2006; Suslow et al., 2010). A recent study has shown that native American English listeners with greater implicit bias toward making Asian-to-foreign and Caucasian-to-American associations experienced greater relative difficulties in transcribing English sentences in background noise, which were produced by native Korean speakers than that produced by native English speakers. This relationship between racial bias and FAS intelligibility was only observed when the auditory stimuli were paired with video recordings of the speakers producing the sentences (Figure 1; Yi et al., 2013). In spite of the novelty of the finding, did not reach a conclusive implication of the behavioral results, but rather cautiously suggesting that the listener bias likely led to altered incorporation of visual cues which are beneficial for enhancing speech intelligibility in adverse listening situations (Sumby and Pollack, 1954; Grant and Seitz, 2000). The precise neural mechanism underlying the relationship between listener bias and FAS perception remains unclear.

In this fMRI study, monolingual native English speakers (*N* = 19) were presented with English sentences produced by native English or native Korean speakers in an MR scanner. The sentences were presented either along with video recordings of the speakers producing the sentences (“audiovisual modality”) or without (“audio-only modality”). A rapid event-related design was used to acquire functional images. This setup allowed us to independently estimate BOLD responses to stimulus presentation and motor response on a trial-by-trial basis. Outside the scanner, the participants performed an IAT which was designed to measure the extent of the association between Asian faces and foreignness. Whole brain analyses were conducted to test the prediction that FAS perception would involve increased activation in the superior temporal cortex and the articulatory-phonological network, consistent with previous research on foreign phonemes processing (Golestani and Zatorre, 2004; Wilson and Iacoboni, 2006), speech intelligibility processing (Adank, 2012), and categorical perception in the inferior frontal gyrus (Myers et al., 2009; Rauschecker and Scott, 2009; Lee et al., 2012). ROI analyses were conducted to test whether the degree of implicit association between Asian faces and foreignness would be associated with modifications in the signal-to-phonology mapping process. For this purpose, the ROI analysis was restricted to the primary auditory cortex, involved in auditory input processing, and the inferior frontal gyrus, involved in phonological processing. Previous neuroimaging studies utilizing IAT as a covariate have consistently shown positive correlation between the IAT scores and the neural response to the dispreferred stimuli, which has led us to hypothesize that higher IAT scores (stronger Asian-Foreign and Caucasian-American association) would be associated with greater BOLD response to FAS, especially in the audiovisual modality.

## Materials and methods

### Materials

#### Participants

Nineteen young adults (age range: 18–35; 11 female) were recruited from the Austin community. All participants passed a hearing-screening exam (audiological thresholds <25 dB HL across octaves from 500 to 4000 Hz), had normal or corrected to normal vision, and self-reported to be right-handed. Potential participants were excluded if their responses to a standardized language history questionnaire revealed significant exposure to any language other than American English (LEAP-Q; Marian et al., 2007). Data from one participant (male) were excluded from all analysis due to detection of a structural anomaly. All recruitment and participation procedures were conducted in adherence to the University of Texas at Austin Institutional Review Board.

#### Audiovisual speech stimuli

Four native American English (2 female) and four native Korean speakers (2 female) produced 80 meaningful sentences with four keywords each (e.g., “the GIRL LOVED the SWEET COFFEE”; Calandruccio and Smiljanic, 2012). The speakers were between 18 and 35 years of age. The speakers were instructed to read text provided on the prompter as if they were talking to someone familiar with their voice and speech patterns. The NAS stimuli had been rated to be 96.2% native-like, and the FAS stimuli had been rated to be 20.7% native-like (converted from a 1-to-9 Likert scale; Yi et al., 2013). Twenty non-overlapping sentences from each speaker was selected, resulting in 80 sentence stimuli used in the experiment. The video track was recorded using a Sony PMW-EX3 studio camera, and the audio track was recorded with an Audio Technica AT835b shotgun microphone placed on a floor stand in front of the speaker. Camera output was processed through a Ross crosspoint video switcher and recorded on an AJA Pro video recorder. The recording session was conducted on a sound-attenuated sound stage at The University of Texas at Austin. The raw video stream was exported using the following specifications. Codec: DV Video (dvsd); resolution: 720 × 576; frame rate: 29.969730 (Figure 1). The raw audio stream was RMS amplitude normalized to 62 dB SNL and exported using the following specifications. Codec: PCM S16 LE (araw); mono; sample rate: 48 kHz; 16 bits per sample.

#### IAT

Ten young adult Asian (5 female) and 10 Caucasian (5 female) face images were used for Caucasian vs. Asian face categories (Minear and Park, 2004). All face images had been edited to exclude hair, face contour, ear, and neck information, then rendered into grayscale with constant luminosity (Goh et al., 2010). Public domain images of 10 iconic American scenes (Grand Canyon, Statue of Liberty, Wrigley Field, Golden Gate Bridge, Pentagon, Liberty Bell, White House, Capitol, New York Central Park, Empire State Building) and 10 non-American foreign scenes (Eiffel Tower, Pyramids, Angkor Wat, London Bridge, Brandenburg Gate, Stonehenge, Great Wall of China, Leaning Tower of Pisa, Sydney Opera House, Taj Mahal) were obtained online and used for American vs. Foreign scene categories. No scene image contained face information. All images were cropped to a square proportion. The stimuli and the design used for the IAT were identical to those used in our previous study (Figure 1; Yi et al., 2013).

### Methods

#### Scan parameters

The participants were scanned via the Siemens Magnetom Skyra 3T MRI scanner at the Imaging Research Center of the University of Texas at Austin. High-resolution whole-brain T1-weighted anatomical images were obtained via MPRAGE sequence (*TR* = 2.53 s; *TE* = 3.37 ms; FOV = 25 cm; 256 × 256 matrix; 1 × 1 mm voxels; 176 axial slices; slice thickness = 1 mm; distance factor = 0%). T2^*^-weighted Whole-brain blood oxygen level dependent (BOLD) images were obtained using a gradient-echo multi-band EPI pulse sequence (flip angle = 60°; *TR* = 1.8 s; 166 repetitions; *TE* = 30 ms; FOV = 25 cm; 128 × 128 matrix; 2 × 2 mm voxels; 36 axial slices; slice thickness = 2 mm; distance factor = 50%) using GRAPPA with an acceleration factor of 2. Three hundred and thirty-four time points were collected, resulting in the scanning duration of approximately 10 min. This was a part of a larger scanning protocol which lasted for approximately 1 h for each participant.

#### fMRI task

Participants were instructed to listen to the recorded sentences and rate the clarity of each one. After the presentation of each stimulus, a screen prompting the response was presented, upon which the participants rated the clarity of the stimulus by pressing one of the four buttons on the button boxes, ranging from 1 (not clear) to 4 (very clear). This was done to ensure that participants were attending to the presentation of the stimuli. The audio track for the sentences were presented auditorily via MR-compatible insert earphones (ER30; Etymotic Research), and the visual track was presented via projector visible by an in-scanner mirror. The stimuli were spoken by native English or native Korean speakers. There were two experimental conditions: an audio-only condition where only the acoustic signals were presented with a fixation cross being displayed, and an audiovisual condition where the video recordings of the talkers' faces producing the sentences were also presented. All sentences were presented only once in a single session without breaks. Therefore, the 80 sentences were subdivided into 20 sentences per each of the four conditions (native with visual cues; native without visual cues; nonnative with visual cues; nonnative without visual cues). We used a rapid event related design with jittered interstimulus intervals of 2–3 s. The order of the stimuli followed a pseudorandom sequence predetermined to avoid consecutive runs of stimuli of a given condition.

#### IAT

Following the fMRI acquisition session, IAT was conducted outside the scanner in a soundproof testing room. The IAT procedures were identical to those used in our previous study (Yi et al., 2013). For each trial, a face or scene stimulus was displayed on the screen. The face stimuli differed from the main task in the scanner in that they were still images unrelated to sentence production. In the congruous category condition, participants had to press a key on the keyboard when they saw a Caucasian face or an American scene, and a different key for an Asian face or a Foreign scene. In the incongruous category condition, participants had to press a key for a Caucasian face or a Foreign scene, and a different key for an Asian face or an American scene (Devos and Banaji, 2005). Participants were instructed to respond as quickly as possible without sacrificing accuracy. Each condition was presented twice with the key designations switched in a randomized order. These yielded four test blocks. Four practice blocks were included prior to the test blocks, in which only scenes or faces were presented. An incorrect response led to a corrective feedback of “Error!” (Greenwald et al., 2003).

### Analyses

#### IAT

In a standalone analysis, a linear mixed effects analysis (Bates et al., 2012) was run with the response times in milliseconds as the dependent variable to directly quantify the delayed response times due to the incongruous association. The category condition (congruous vs. incongruous) and the neural index were entered as the fixed effects to measure the delay effect of face-scene pairings incongruent with the implicit association. By-subject random intercepts were included. The optimizer was set to BOBYQA (Powell, 2009). Individual IAT scores were calculated following the standard guidelines (Greenwald et al., 2003). Trials with response times longer than 10,000 ms or shorter than 400 ms were excluded. Response times for incorrect trials were replaced by the mean of the response times for correct trials within the same block, increased by 600 ms. The average response time discrepancies across the two pairs of congruous vs. incongruous blocks were divided by the standard deviation of response times in the two blocks. These two discrepancy measures were averaged to yield in the final IAT score, which was used as a covariate in other analyses.

#### Clarity rating

Clarity ratings for all sentences from each participant were entered as the dependent variable, after being mean-centered to 0, in a linear mixed effects analysis. In order to counteract different clarity criteria across the participants, the model was corrected for by-participant random intercepts. In the first analysis, the fixed effects included the accent and modality of the stimuli, the individual IAT measures, and the ensuing interactions. The optimizer was set to BOBYQA (Powell, 2009).

#### fMRI preprocessing

fMRI data were analyzed using FMRIB's Software Library Version 5.0 (Smith et al., 2004; Woolrich et al., 2009; Jenkinson et al., 2012). BOLD images were motion corrected using MCFLIRT (Jenkinson et al., 2002). All images were brain-extracted using BET (Smith, 2002; Jenkinson et al., 2005). Registration to the high-resolution anatomical image (*df* = 6) and the MNI 152 template (*df* = 12; Grabner et al., 2006a) was conducted using FLIRT (Jenkinson and Smith, 2001; Jenkinson et al., 2002). Six separate block-wise first-level analysis were run within-subject. The following pre-statistics processing were applied; spatial smoothing using a Gaussian kernel (FWHM = 5 mm); grand-mean intensity normalization of the entire 4D dataset by a single multiplicative factor; highpass temporal filtering (Gaussian-weighted least-squares straight line fitting, with sigma = 50.0 s). Each event was modeled as an impulse convolved with a canonical double-gamma hemodynamic response function (phase = 0 s). Motion estimates were modeled as nuisance covariates. Temporal derivative of each event regressor, including the motion estimates, was added. Time-series statistical analysis was carried out using FILM with local autocorrelation correction (Smith et al., 2004). The event regressors consisted of stimulus, response screen, and clarity response. The stimulus regressors were subdivided into accent (native vs. foreign) and modality (audiovisual vs. audio-only) conditions. The missed trials were separately estimated as nuisance variables. Three sets of *t*-test contrast pairs were tested, which examined modality (audiovisual – audio-only; audio-only – audiovisual), accent (native-accented – foreign-accented; foreign-accented – native-accented), and the interaction effects (audiovisual native – audiovisual foreign – audio-only native + audio-only foreign; audiovisual foreign – audiovisual native – audio-only foreign + audio-only native).

#### Whole brain analysis

Group analysis was performed for each contrast using FLAME1 (Woolrich et al., 2009). To correct for multiple comparisons, post-statistical analysis was performed using randomize in FSL to run permutation tests (*n* = 50,000) for the GLM and yield in threshold-free cluster enhancement (TFCE) estimates of statistical significance. The corresponding family-wise error corrected *p*-values are presented in the results (Freedman and Lane, 1983; Kennedy, 1995; Bullmore et al., 1999; Anderson and Robinson, 2001; Nichols and Holmes, 2002; Hayasaka and Nichols, 2003). The results are presented in the Table 1.

**Table 1:** **Whole brain analysis results for the contrasts of interest**.

**Contrast**	**Regions**	***x* (mm)**	***y* (mm)**	***z* (mm)**	**Voxels**
**MODALITY EFFECT**					
(a) Audiovisual – audio-only	Bilateral occipital cortex; Bilateral fusiform gyri; L posterior superior temporal gyrus; Bilateral posterior middle temporal gyri	38	−46	−22	14108
	R thalamus	16	−32	2	208
	L thalamus	−26	−30	−4	188
	R amygdala	20	−4	−14	16
	R temporal pole	56	6	−22	13
(b) Audio-only – audiovisual	R supeior parietal lobule; R somatosensory cortex; R supramarginal gyrus	34	−38	50	1864
	R superior frontal gyrus; R primary motor cortex	24	4	64	804
	L superior parietal lobule; R somatosensory cortex	−38	−36	42	330
	L middle frontal gyrus	−32	42	30	315
	L superior frontal gyrus	−26	4	56	177
	R middle frontal gyrus	30	36	22	82
**ACCENT EFFECT**					
(c) Native – foreign	R posterior middle temporal gyrus; R posterior inferior temporal gyrus; R angular gyrus; R supramarginal gyrus	64	−46	26	379
(d) Foreign – native	Paracingulate gyrus	4	24	34	904
	R motor cortex, R superior parietal lobule; R somatosensory cortex	34	−52	62	868
	L insular cortex	−40	14	8	76
	R superior frontal gyrus	20	−2	60	68
	R insular cortex	32	20	−6	50
	L inferior frontal gyrus	−52	10	10	42
	L insular cortex	−26	24	0	31
	R insular cortex	42	14	8	13
Modality by accent interaction	n.s.				

Clusters are based on the p < 0.025 threshold as well as the size criterion of 10 voxels.

#### ROI analysis

The ROIs were anatomically defined as the left and right primary auditory cortices (combination of Te 1.0, 1.1, and 1.2; Morosan et al., 2001) and the left inferior frontal gyrus (Brodmann area 44; Amunts et al., 1999) using the Jülich histological atlas (threshold = 25%; Eickhoff et al., 2005, 2006, 2007). Percent changes in BOLD responses for the stimuli in four conditions (native-accented with faces; native-accented without faces; foreign-accented with faces; foreign-accented without faces) were calculated by first linearly registering the ROIs to the individual BOLD spaces using FLIRT with the appropriate transformation matrices generated from the first level analysis and nearest neighbor interpolation (Jenkinson and Smith, 2001; Jenkinson et al., 2002). Then, the parameter estimate images were masked for the transformed ROIs, multiplied by height of the double gamma function for the stimulus length of 2 s (0.4075), converted into percent scale, divided by mean functional activation, and averaged within the ROI, using fslmaths (Mumford, 2007). The percent signal change was entered as the dependent variable in a linear mixed effects analysis. In the mixed effects analysis, the fixed effects included the accent (native vs. foreign), face (faces vs. no faces), individual IAT values and their interaction terms. The model was corrected for by-participant random intercepts (Bates et al., 2012). The optimizer was set to BOBYQA (Powell, 2009).

## Results

### Behavioral results

#### Clarity ratings

The overall mean clarity rating was 2.94 (*SD* = 1.09). The mean clarity rating for the NAS was 3.28 (*SD* = 1.10) in the audio-only condition and 3.34 (*SD* = 1.06) in the audiovisual condition, while the rating for the FAS was 2.55 (*SD* = 0.96) in the audio-only condition and 2.57 (*SD* = 0.96) in the audiovisual condition. In this analysis, the fixed effects of modality, accent, the IAT scores, and their interaction terms were included as fixed effects for the dependent variable of clarity ratings for each sentence, which was mean-centered to 0. The three-way interaction was not significant, which was excluded in the final model. The accent effect was significant, *b* = −0.89, *SE* = 0.095, *t* = −9.36, *p* < 0.0001, 95% CI [−1.08, −0.70], indicating that FAS was perceived to be less clear than NAS. The accent by IAT interaction was significant, *b* = 0.33, *SE* = 0.15, *t* = 2.13, *p* = 0.034, 95% CI [0.026, 0.63], indicating that higher IAT values were more associated with higher perceived clarity ratings for the FAS relative to NAS. The intercept was significant, *b* = 0.97, *SE* = 0.39, *t* = 2.45, *p* = 0.024, 95% CI [0.15, 1.78]. The modality effect was not significant, *b* = −0.015, *SE* = 0.094, *t* = −0.16, *p* = 0.88, 95% CI [−0.20, 0.17], failing to provide evidence that perceived clarity was modified by the availability of visual cues. This is in contrast to the extensive previous literature that have indicated the intelligibility benefit from the audiovisual modality (Sumby and Pollack, 1954; Macleod and Summerfield, 1987; Ross et al., 2007). We ascribe this null finding to the task properties which did not require the participants to actively decipher the sentences, but only to rate their clarity (Munro and Derwing, 1995a). The IAT effect was not significant, *b* = −0.34, *SE* = 0.70, *t* = −0.49, *p* = 0.63, 95% CI [−1.79, 1.10]. The modality by accent interaction was not significant, *b* = −0.054, *SE* = 0.077, *t* = −0.71, *p* = 0.48, 95% CI [−0.20, 0.096]. The modality by IAT interaction was not significant, *b* = 0.15, *SE* = 0.15, *t* = 0.97, *p* = 0.33, 95% CI [−0.15, 0.45]. These results altogether suggest that FAS is perceived to be less clear by the listeners. Participants with higher IAT scores, i.e., those who were more likely to implicitly associate East Asian faces with foreignness, have decreased tendency to perceive FAS to be unclear, compared to NAS.

#### IAT

The overall mean response time was 948 ms (*SD* = 586 ms). The mean RT was 824 ms (*SD* = 408 ms) in the congruous condition, and 1073 ms (*SD* = 700 ms) in the incongruous condition. One fixed effects term was included in the model: incongruity of the stimuli pairing. The intercept was significant, *b* = 823.83, *SE* = 50.92, *t* = 19.50, *p* < 0.0001, 95% CI [718.84, 928.81], showing approximately 820 ms baseline response time. The incongruity effect was significant, *b* = 249.24, *SE* = 19.90, *t* = 12.52, *p* < 0.0001, 95% CI [210.22, 288.27], suggesting that incongruous stimuli pairing delayed each response by approximately 250 ms. The mean IAT score was calculated to be 0.51 (*SD* = 0.25), indicating a general trend of implicit bias toward making the Asian-Foreign association.

### fMRI results

#### Audio-only vs. Audiovisual

**Figure 2:**
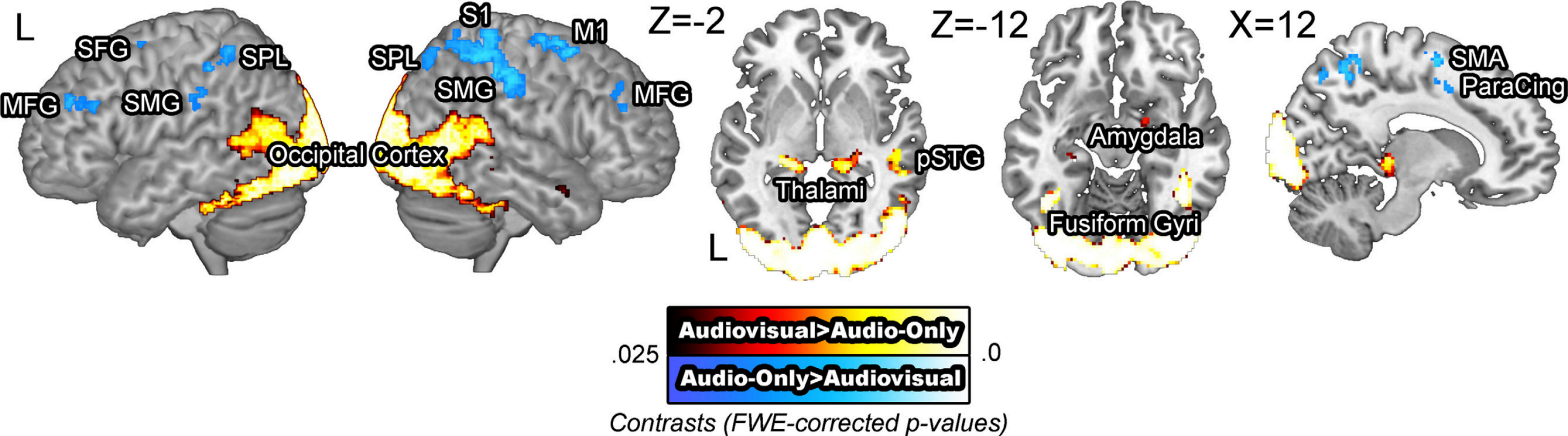
**BOLD signals in the audio-only vs. audiovisual comparison**. The [audiovisual – audio-only] contrast revealed extensive activity in the occipital cortex and the bilateral posterior middle temporal gyri. The [audio-only – audiovisual] contrast revealed activity in the right middle frontal gyrus, right motor and somatosensory areas and the superior parietal lobule.

BOLD signals were compared across the audiovisual and audio-only stimuli. The [audiovisual – audio-only] contrast revealed extensive activity in the occipital cortex, as the visual information in the faces required computations in the visual modality. Activity in the bilateral middle temporal gyri, left posterior superior temporal gyrus, and the right temporal pole was also observed, presumed to reflect integrative effort of the visual cues available in the facial stimuli (Sams et al., 1991; Möttönen et al., 2002; Pekkola et al., 2005). The [audio-only – audiovisual] contrast revealed activity in the bilateral superior and middle frontal gyri, right motor and somatosensory cortices, and the bilateral supramarginal gyri (Figure 2). The increased activation in the motor and somatosensory areas for audio-only speech than for audiovisual speech is in contrast to previous research that has shown the opposite pattern (Skipper et al., 2005). It is possible that the absence of visual cues induced more effortful processing in these areas. The activity in these regions is presumed to reflect the necessity of additional computation in the speech processing network.

#### Native- vs. Foreign-accented speech

**Figure 3:**
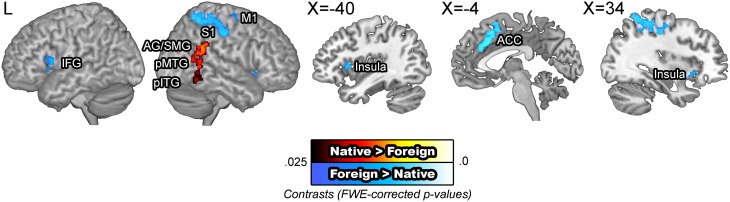
**BOLD signals in the native vs. foreign accented speech comparison**. The [native – foreign] contrast revealed greater activity in the right angular gyrus and the posterior middle temporal gyrus. The [foreign – native] contrast revealed greater activity along the bilateral superior temporal gyri, anterior cingulate cortex, and the bilateral caudate nuclei. The articulatory network, encompassing the bilateral inferior frontal gyri, insula, and the right motor cortex were also additionally activated.

BOLD signals were compared across the speaker accent. The [native – foreign] contrast revealed greater activity in the right angular gyrus, supramarginal gyrus, the posterior middle, and inferior temporal gyri. Supramarginal gyri have been suggested to be involved in making phonological decisions, which in the context of this study is presumed to reflect improved phonological processing for NAS than for FAS (Hartwigsen et al., 2010). The [foreign – native] contrast revealed greater activity in the motor cortex, somatosensory cortex, inferior frontal gyrus, insula, and the anterior cingulate cortex. These areas have been previously indicated to be additionally recruited for perception of foreign phonemes (Golestani and Zatorre, 2004; Wilson and Iacoboni, 2006) or distorted speech (Adank, 2012). The omission of the superior temporal areas are significant, which run counter to our initial hypothesis regarding increased computational demand due to unfamiliar auditory input. A potential interpretation of this null finding is that the activity in the superior temporal cortex was more variable than that in the motor and frontal areas, an idea which was tested in the subsequent ROI analysis (Figure 3). The modality by accent interaction contrasts did not yield significant results.

#### ROI analyses

**Figure 4:**
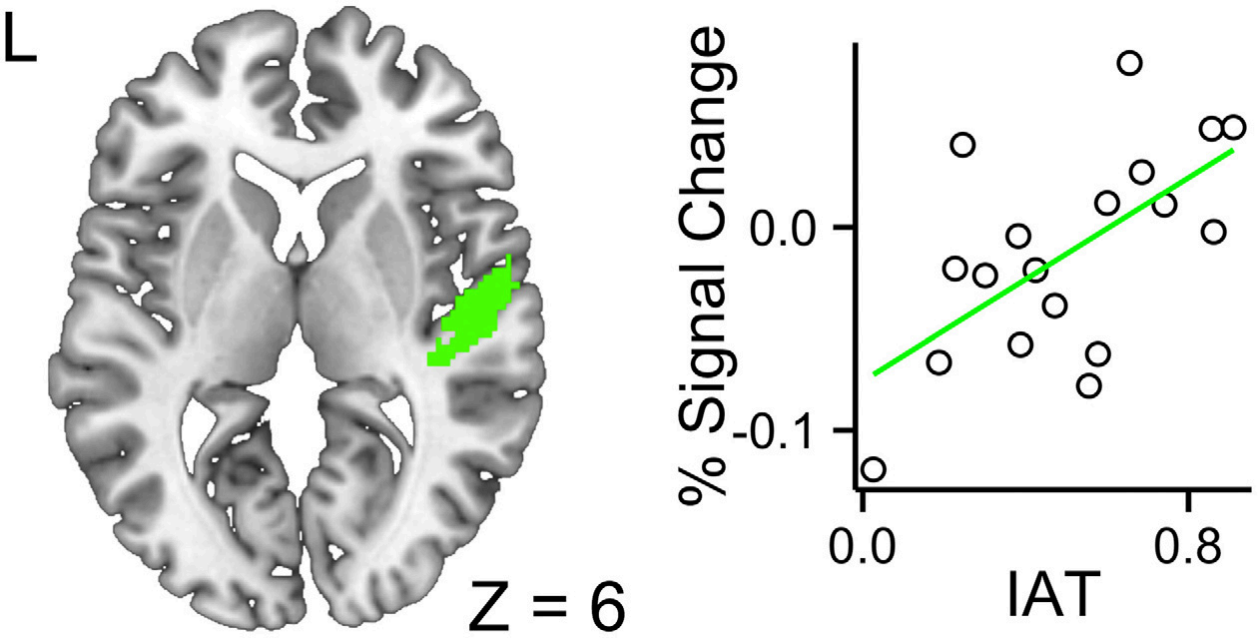
**Higher a given participant's IAT score, greater the BOLD response for the interaction contrast between foreign-accented speech and the availability of faces in the right primary auditory cortex**. This indicated that participants with higher IAT scores required additional processing resources for foreign-accented speech with faces.

The ROI analyses were constrained to the left and right primary auditory cortices and the left inferior frontal gyrus. The fixed effects included accent (foreign- vs. native-accented speech), modality (audiovisual vs. audio-only), IAT scores, and their interaction terms. In the left primary auditory cortex, no three-way or two-way interactions were significant, leaving the model with only three main effects of accent, modality and IAT to be considered. The modality effect was significant, *b* = −0.047, *SE* = 0.014, *t* = −3.34, *p* = 0.0016, 95% CI [−0.075, −0.020], suggesting that the audiovisual stimuli reduced computational demand in this region, relative to the audio-only stimuli. The accent effect was not significant, *b* = 0.010, *SE* = 0.014, *t* = 0.71, *p* = 0.48, 95% CI [−0.018, 0.038]. The IAT effect was not significant, *b* = −0.27, *SE* = 0.20, *t* = −1.31, *p* = 0.21, 95% CI [−0.66, 0.13]. The intercept was significant, *b* = 0.45, *SE* = 0.11, *t* = 3.89, *p* = 0.0013, 95% CI [0.22, 0.67]. In the right primary auditory cortex, the three-way interaction across modality, accent, and IAT was significant, *b* = 0.25, *SE* = 0.11, *t* = 2.26, *p* = 0.028, 95% CI [0.042, 0.46], suggesting that higher IAT scores were associated with increased response to FAS with faces (Figure 4). The interaction between accent and modality was significant, *b* = −0.15, *SE* = 0.062, *t* = −2.43, *p* = 0.019, 95% CI [−0.27, −0.34], suggesting that the decreased neural efficiency due to FAS was ameliorated by the availability of faces. The accent effect was significant, *b* = 0.090, *SE* = 0.044, *t* = 2.04, *p* = 0.047, 95% CI [0.0070, 0.17], suggesting that FAS increased the computational demand in this region. The intercept was significant, *b* = 0.32, *SE* = 0.12, *t* = 2.57, *p* = 0.020, 95% CI [0.077, 0.55]. The accent by IAT interaction was not significant, *b* = −0.061, *SE* = 0.078, *t* = −0.78, *p* = 0.44, 95% CI [−0.21, 0.087]. The IAT by modality interaction was not significant, *b* = −0.13, *SE* = 0.078, *t* = −1.65, *p* = 0.11, 95% CI [−0.28, 0.018]. The modality by accent interaction was not significant, *b* = −0.15, *SE* = 0.062, *t* = −2.43, *p* = 0.019, 95% CI [−0.27, −0.034]. The IAT main effect was not significant, *b* = −0.23, *SE* = 0.22, *t* = −1.03, *p* = 0.32, 95% CI [−0.65, 0.20]. The modality main effect was not significant, *b* = 0.011, *SE* = 0.044, *t* = 0.25, *p* = 0.81, 95% CI [−0.072, 0.094]. The intercept was significant, *b* = 0.32, *SE* = 0.12, *t* = 2.57, *p* = 0.020, 95% CI [0.077, 0.55].

In the left inferior frontal gyrus, no three-way or two-way interactions were significant, leaving the model with only three main effects of accent, modality and IAT to be considered. The accent effect was significant, *b* = 0.058, *SE* = 0.018, *t* = 3.15, *p* = 0.0028, 95% CI [0.022, 0.094], suggesting that FAS increased computational demand in this region. The IAT effect was not significant, *b* = −0.37, *SE* = 0.20, *t* = −1.83, *p* = 0.086, 95% CI [−0.76, 0.024]. The modality effect was not significant, *b* = −0.026, *SE* = 0.018, *t* = −1.41, *p* = 0.16, 95% CI [−0.062, 0.010]. The intercept was not significant, *b* = 0.12, *SE* = 0.11, *t* = 1.02, *p* = 0.32, 95% CI [−0.11, 0.34]. In the right inferior frontal gyrus, no three-way or two-way interactions were significant, leaving the model with only three main effects of accent, modality, and IAT to be considered, The accent effect was not significant, *b* = 0.0080, *SE* = 0.019, *t* = 0.43, *p* = 0.67, 95% CI [−0.029, 0.045]. The IAT effect was not significant, *b* = −0.36, *SE* = 0.19, *t* = −1.89, *p* = 0.077, 95% CI [−0.74, 0.011]. The modality effect was not significant, *b* = −0.0090, *SE* = 0.019, *t* = −0.48, *p* = 0.63, 95% CI [−0.046, 0.028]. The intercept was not significant, *b* = 0.12, *SE* = 0.11, *t* = 1.10, *p* = 0.29, 95% CI [−0.093, 0.33].

## Discussion

Listening to FAS can be challenging compared to NAS. This difficulty can be partly attributed to a demanding process of mapping somewhat unreliable incoming signals to phonology. We hypothesized that FAS perception will require additional spectrotemporal analysis of the acoustic signal and place a greater demand on the phonological processing network. We therefore predicted increased functional activity in the superior temporal cortex and the inferior frontal gyrus, insula, and the motor cortex (Hickok and Poeppel, 2007; Rauschecker and Scott, 2009; Adank and Devlin, 2010; Adank et al., 2012a,b). Furthermore, FAS perception is additionally modulated by listeners' underlying implicit bias (Greenwald et al., 1998, 2009; Mcconnell and Leibold, 2001; Bertrand et al., 2005; Devos and Banaji, 2005; Kinoshita and Peek-O'leary, 2005; Yi et al., 2013). Thus, we hypothesized that individual variability in implicit Asian-foreign association will be associated with functional activity during early spectrotemporal analysis in the primary auditory cortex or for later, more categorical processing in the inferior frontal gyrus (Hickok and Poeppel, 2007; Rauschecker and Scott, 2009).

### Increased computational demand for foreign-accented speech processing

Relative to native speech, FAS was associated with increased BOLD response in the bilateral superior temporal cortices, potentially reflecting increased computational demand on these regions. The anterior and posterior portions of the superior temporal cortex have been associated with spectrotemporal analysis of the speech signal (Hickok and Poeppel, 2007), as well as with speech intelligibility processing (Scott et al., 2000; Narain et al., 2003; Okada et al., 2010; Abrams et al., 2013). While these previous studies have observed increased activation of the superior temporal cortex for intelligible speech compared to unintelligible acoustically complex stimuli, we found increased activation for the FAS stimuli than for the NAS stimuli, although FAS is less intelligible than NAS (Yi et al., 2013). This contradiction is resolved by considering the nature of comparisons involved in previous neuroimaging studies examining mechanisms underlying speech intelligibility. Both native- and FAS used in the current study have semantic and syntactic content which are absent in non-speech stimuli used as control in the previous studies (e.g., spectrally-rotated speech), and both functions have been suggested to occur within the superior temporal cortex (Friederici et al., 2003). The superior temporal cortex is a large region with possibly multiple functional roles in processing information in the speech signal. A direct recording study has shown that speech sound categorization is represented in the posterior superior temporal cortex (Chang et al., 2010), while a direct stimulation study had indicated the role of anterior superior temporal cortex in comprehension but not auditory perception (Matsumoto et al., 2011).

We found that presentation of FAS was associated with greater activity in the articulatory-phonological network, encompassing bilateral inferior frontal gyri, insula, and the right motor cortex. The inferior frontal gyrus is thought to be responsible for mapping auditory signals onto articulatory gestures (Myers et al., 2009; Lee et al., 2012; Chevillet et al., 2013). It has been suggested that the role of the inferior frontal gyrus is defined by the linkage between motor observation and imitation, which allows for abstraction of articulatory gestures from the auditory signals, along with the motor cortex and the insula (Ackermann and Riecker, 2004, 2010; Molnar-Szakacs et al., 2005; Pulvermüller, 2005; Pulvermüller et al., 2005, 2006; Skipper et al., 2005; Galantucci et al., 2006; Meister et al., 2007; Iacoboni, 2008; Kilner et al., 2009; Pulvermüller and Fadiga, 2010). On the other hand, both fMRI and transcranial magnetic stimulation (TMS) studies have indicated a functional heterogeneity within the inferior frontal cortex, which includes semantic processing (Homae et al., 2002; Devlin et al., 2003; Gough et al., 2005). The FAS and NAS stimuli had been controlled for syntactic, semantic, and phonological complexity (Calandruccio and Smiljanic, 2012). Since the task for each stimulus had also been identical (clarity rating), the increased activation across the speech processing network—including the superior temporal cortex and the articulatory network—during FAS perception is interpreted to reflect decreased neural efficiency for FAS processing (Grabner et al., 2006b; Rypma et al., 2006; Neubauer and Fink, 2009).

### Implicit asian-foreign association associated with early spectrotemporal analysis

Previous behavioral studies have shown that FAS perception is modulated not only by the signal-driven factors, but also by the listener-driven factors. These listener factors can include listeners' familiarity and experience with the talkers (Bradlow and Pisoni, 1999) or language experience (Bradlow and Pisoni, 1999; Bent and Bradlow, 2003). Multiple studies have shown that listeners are also sensitive to the information regarding talker properties (Campbell-Kibler, 2010; Drager, 2010), either through explicit labels (Niedzielski, 1999; Hay et al., 2006a; Floccia et al., 2009) or facial cues (Strand, 1999; Hay et al., 2006b; Drager, 2011; McGowan, 2011; Yi et al., 2013). Listeners vary in their susceptibility to these talker cues (Hay et al., 2006b), which can override their explicit knowledge (Hay et al., 2006a). Accordingly, listeners' implicit association between faces and foreignness (Greenwald et al., 1998, 2009; Mcconnell and Leibold, 2001; Bertrand et al., 2005; Devos and Banaji, 2005; Kinoshita and Peek-O'leary, 2005) modulates FAS perception only when the faces are present, through a neural mechanism hitherto unknown (Yi et al., 2013). In this study, the IAT was used to measure the degree of listener bias in which the East Asian faces are associated with foreignness of the speakers (Greenwald et al., 1998, 2009; Mcconnell and Leibold, 2001; Bertrand et al., 2005; Devos and Banaji, 2005; Kinoshita and Peek-O'leary, 2005). Previous fMRI studies that have used IAT as a covariate have consistently showed a pattern in which higher measures of implicit association are associated with higher activation in various neural areas for dispreferred stimuli (Richeson et al., 2003; Krendl et al., 2006; Luo et al., 2006; Suslow et al., 2010).

Examining the connection between FAS perception and listener bias, we found that listeners' implicit Asian-foreign association was reflected in the functional activity in the right primary auditory cortex. Participants with higher IAT scores showed greater activity in the primary auditory cortex for Korean-accented sentences when audiovisual information was presented. The primary auditory cortex is the site for early spectrotemporal analysis for the speech signal, sensitive to acoustic properties of the signal (Strainer et al., 1997), as well as task demands (Fritz et al., 2003, 2005), attention (Jäncke et al., 1999), context (Javit et al., 1994), and training effects (Recanzone et al., 1993). In contrast, IAT scores were not associated with the activity in the inferior frontal gyrus. Past findings indicated that individual listeners' perceived talker properties from pictorial stimuli differentially modulate the perceptual experience (Hay et al., 2006b). In the case of FAS, the presentation of race-matched faces enhanced perception of Chinese-accented English speech (McGowan, 2011), and the individual variability in implicit Asian-foreign association predicted Korean-accented speech intelligibility (Yi et al., 2013). The present findings suggest that the listeners' implicit bias for associating Asian speakers with foreignness may be related to the early neural processing for FAS, specifically low-level spectrotemporal analysis of the acoustic properties of the signal.

### Limitations and future directions

In this study, Korean-accented speech was used as the proxy for FAS. Accordingly, all foreign-accented talkers appeared East Asian. In order to extend our results to the general phenomenon of FAS perception, we propose a multifactorial design in future studies where, in addition to the stimuli produced by Caucasian native speakers and Asian nonnative speakers, those by Asian native speakers and Caucasian nonnative speakers are incorporated into the study design. Also, additional explicit questionnaire on listener experience and exposure to foreign-accented stimuli could be collected to augment our understanding of the complex nature in which underlying listener biases modulate speech perception. Finally, we acknowledge that the current study did not incorporate parametric variations on the intelligibility or accentedness of the FAS stimuli. Therefore, it is impossible to determine whether the increased BOLD response in the speech processing areas and the anterior cingulate cortex reflects increased difficulty in comprehension or the degree of perceived foreign accent *per se* (Peelle et al., 2004; Wong et al., 2008).

## Conclusions

In this study, we presented evidence of increased computational demand for FAS perception. Changes in the reduced neural efficiency for FAS processing was associated with the variability in the underlying listener biases (Yi et al., 2013). These results suggest that implicit racial association is associated with early neural response to FAS. Future studies on speech perception should examine the contribution of visual cues and listener implicit biases in order to obtain a more comprehensive understanding of the phenomenon of FAS processing.

### Conflict of interest statement

The authors declare that the research was conducted in the absence of any commercial or financial relationships that could be construed as a potential conflict of interest.
